# Modification of a single-molecule AFM probe with highly defined surface functionality

**DOI:** 10.3762/bjnano.5.221

**Published:** 2014-11-14

**Authors:** Fei Long, Bin Cao, Ashok Khanal, Shiyue Fang, Reza Shahbazian-Yassar

**Affiliations:** 1 Department of Mechanical Engineering-Engineering Mechanics, Michigan Technological University, 1400 Townsend Drive, Houghton, Michigan, USA; 2 Department of Chemistry, Michigan Technological University, 1400 Townsend Drive, Houghton, Michigan, USA

**Keywords:** atomic force microscopy, click reaction, force spectroscopy, single molecule modification

## Abstract

Single-molecule force spectroscopy with an atomic force microscope has been widely used to study inter- and intramolecular interactions. To obtain data consistent with single molecular events, a well-defined method is critical to limit the number of molecules at the apex of an AFM probe to one or to a few. In this paper, we demonstrate an easy method for single-molecule probe modification by using the Cu-catalyzed alkyne–azide cycloaddition reaction. Excess terminal alkynes were covalently attached to the probe, and a bi-functional molecule containing an azide at one end and a carboxylic acid at the other was dissolved in the reaction solution. By simply contacting the probe and the Cu substrate, controlled carboxylation on the probe apex could be achieved, since the ‘click’ reaction requires the co-exist of alkyne, azide and Cu(I). The finite contact area would result in a highly defined surface functionality of the probe down to single molecule level with high reproducibility.

## Introduction

Single-molecule force spectroscopy (SMFS) has become one of the most powerful tools for studying inter- and intramolecular interactions [1]. The information revealed is highly significant for the understanding of the fundamental relationship between molecular structures and their functions [2–5]. Among the SMFS toolbox, atomic force microscopy (AFM) [6] is the most popular one due to high force sensitivity from pico- to nanonewtons, and ease of use under various environment including ambient, aqueous and vacuum [7]. By attaching a single molecule between an AFM probe and a substrate, intramolecular interactions could be studied through stretching the molecule from the entropic form to the extended form. Also, intermolecular interactions could be investigated by attaching one molecule to a probe and the other to a substrate. Examples include the unfolding of proteins [8–9], dissociation of receptors from ligands [10–11], and un-zipping double-stranded RNA and DNA molecules [12]. Despite all the success in these studies, one significant challenge remains in AFM-based SMFS, that is to attach only one or a few molecules to the AFM probes. In previous studies, the most common method depended on non-specific adhesions [13–14], which resulted in uncertainties concerning quantity and location of molecules being studied [1]. To improve the reproducibility of experimental results, covalent attachment methods are preferred. Wong and co-authors attached a single-wall carbon nanotube (SW-CNT) to the tip of an AFM probe [15]. The carboxylic acid group on the open end of the CNT could be further modified with amino and phenyl groups. In this way, the modification of a single-molecule probe with acidic, basic and neutral functionalities could be achieved. Gu and co-authors reported an electrochemical oxidation method for the modification of a single-molecule probe [16]. They applied a bias voltage between an oligo(ethylene glycol) (OEG)-modified AFM probe and an Au film substrate. Carboxylic acid groups were selectively generated at the tip apex through electrochemical oxidation of OEG. The carboxylic acid group was then used to attach the molecules to be studied. All these methods have improved the reproducibility of results from AFM-based single-molecule studies. However, it is not trivial either to attach CNT to an AFM probe, or to adjust bias amplitude/duration in OEG oxidation.

**Figure 1:**
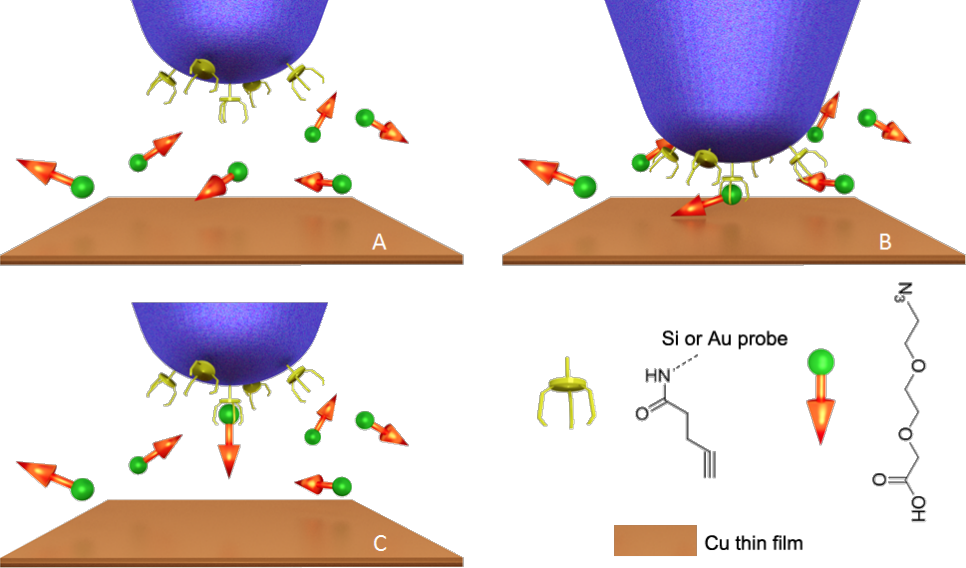
Schematic illustration of the functionalization of a single-molecule probe by using the ‘click’ reaction. (A) An alkyne-modified AFM probe is immersed in a solution of azide/carboxylic acid bi-functional molecules. (B) The probe is brought into contact with a substrate coated with a Cu film for one to two seconds. The Cu(I)-catalyzed alkyne–azide ‘click’ reaction occurs at the contacting area of the probe and substrate. (C) The probe is withdrawn, and single carboxylic acid group is attached to the apex of the probe.

Copper-catalyzed alkyne–azide cycloaddition, which is also called a ‘click’ reaction, has been widely used for surface functionalization and linking of biomolecules [17]. The reaction is compatible with a wide variety of polar functional groups that commonly exist in biomolecules and is highly efficient in various solvents. Chen and co-authors reported the pioneer work of using this ‘click’ reaction to modify AFM probes, taking advantage of the mild reaction condition and simple operation [18]. However, this method is based on a bulk solution and loses the ‘spatial resolution’ in terms of functionality, since the reaction happens anywhere on the probe surface. In this paper, we report a new method for the modification of a single-molecule probe with highly defined surface functionality by using copper-catalyzed alkyne–azide cycloaddition (Figure 1). A probe with a monolayer of terminal alkynes was easily prepared by immersing an amino-functionalized silicon probe in a solution containing 4-pentynoic acid and acetic acid (molar ratio 1:7) through amide bond formation followed by washing and drying (for experimental details see Supporting Information File 1). The probe was then mounted onto an AFM, and brought into contact with a copper-film-coated substrate for 5 to 10 s in a solution containing bi-functional molecules with a carboxylic acid group at one end and an azide group at the other end. Cu(I) was believed to be sparsely scattered over the surface of the copper film. Because the Cu(I)-catalyzed alkyne–azide reaction could only occur at the location where Cu(I), alkyne and azide co-exist, carboxylic acid groups would be attached to the probe during contact. According to DMT model [19], the ~200 pN contact force would result in a contact area within 1 nm in radius. Therefore, the chances for multiple reactions were predicted to be low and single molecular probe modification could be expected. For demonstrating the reproducibility and flexibility, three additional silicon probes and one Au-coated probe were modified under the same conditions.

## Results and Discussion

**Figure 2:**
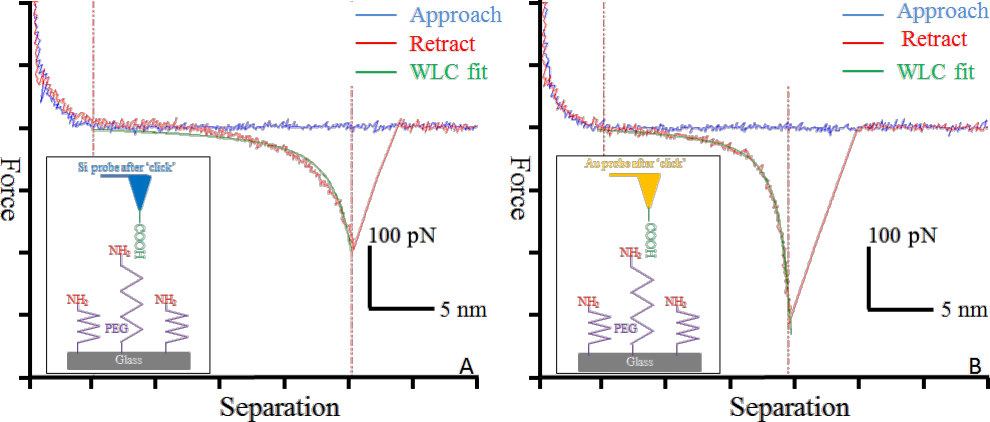
Typical force curves of modified single-molecule (A) Si probes and (B) Au-coated probes interacting with an amino-terminated PEG monolayer on a glass slide. The approach (blue) and retract (red) curves along with worm-like chain model fitting curves (green) are shown, demonstrating the stretching behavior of a single PEG molecule between the probes and the substrate.

To evaluate the results of the modification, a glass slide with a monolayer of amino-terminated poly(ethylene glycol) (PEG) was prepared. PEG was used to reduce the background adhesion force, and as a spacer to better discriminate between specific and non-specific interactions in the force curves [20]. The functionalized probes after ‘click’ modification were ramped over the surface in isopropanol, and more than 1000 force curves were recorded for each of the five probes. Among them, 17–26% showed adhesive interactions appearing at a tip–surface separation greater than zero. Typical force curves obtained were shown in Figure 2A and Figure 2B for the Si probe and the Au-coated probe, respectively. The other force curves showed either no obvious adhesion or only forces at zero separation (due to non-specific interaction). The forces that appear at non-zero tip–surface separations are believed to result from the rupture of a hydrogen bond between the carboxylic acid group on the probe and the amino group on the substrate. Two facts supported this hypothesis. (1) The forces appeared at a tip–surface separation of around 25 nm, which is the contour length of the PEG we used. (2) The force curves fitted well with the worm-like chain (WLC) model [21] with a persistence length of 0.35–0.40 nm, shown as green solid lines in Figure 2. This persistence length agreed well with the extension behavior of PEG molecule [22]. Therefore, it was reasonable to believe that carboxylic groups from the azide molecules had been ‘clicked’ onto the AFM probes through our method.

Three control experiments were carried out to confirm that the ‘click’ reaction indeed occurred at the tip apex of the probes. In these experiments, one of the three components in the ‘click’ reaction (1) the alkyne on the probe, (2) the azide/carboxylic acid bi-functional molecule in the solution, and (3) the Cu film on the substrate, was eliminated. For each control probe, less than 2% of 1000 force curves showed the PEG extension behavior against the amino-terminated PEG monolayer. The significantly lowered probability compared to 17–26% for the probes modified with our method, confirmed that our method can successfully attach carboxylic acid groups to the probe through the ‘click’ reaction.

To further quantify the carboxylic acid groups on a probe, the widely used Poisson statistical method was employed [23–24]. This method assumes the adhesion force is composed of specific interactions, such as hydrogen bonds, and non-specific interactions, such as van der Waals forces,

[1]
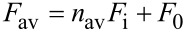


where *F*_av_ is the total average adhesion force, *n*_av_ is the average number of specific interactions, which is hydrogen bonding in our case, *F*_i_ is the magnitude of the specific interaction, and *F*_0_ is non-specific interaction. This method also assumes that the distribution of the average number of interactions at the pull-off point follows a Poisson distribution,

[2]
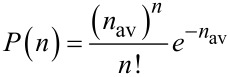


[3]
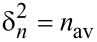


where *P*(*n*) is the possibility of the specific interaction, and 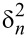 is the variance of the number of interacting bonds. The expectation of a Poisson-distributed random variable is equal to its variance,

[4]
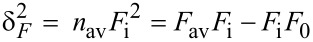


Therefore, by plotting 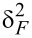 against *F*_av_, a linear relationship is expected with the slope *F*_i_ and intercept –*F*_i_*F*_0_.

**Figure 3:**
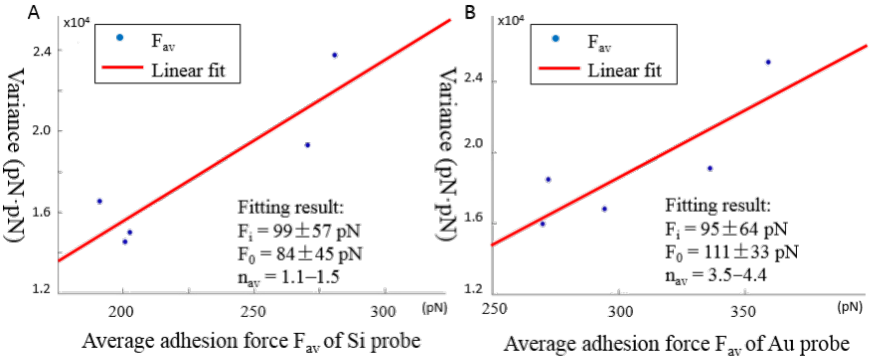
Linear fit of average adhesion force *F*_av_ against its variance by using the Poisson statistical method for (A) the Si probes and (B) the Au-coated probes, respectively. The fitting result is also shown as inset, in which *F*_i_ is the specific interaction, *F*_0_ is the non-specific interaction, and *n*_av_ is the average number of interacting molecules.

The linear fitting results are shown in Figure 3 for one silicon probe and one Au-coated probe. The deduced *F*_i_, *F*_0_ and *n*_av_ are 99 ± 57 pN, 84 ± 45 pN and 1.1–1.5, respectively, for the silicon probe, and 95 ± 64 pN, 111 ± 33 pN, and 3.5–4.4, respectively, for the Au-coated probe. Both probes had similar values of *F*_i_, showing the same nature of the specific hydrogen bond interaction between the carboxylic acid groups on the probe and amino groups on the substrate. These values agreed with single hydrogen bond rupture forces of 50–250 pN reported in the literature [23,25]. The *F*_0_ value of the Au-coated probe was larger than that of the Si probe. This can be attributed to the larger radius of Au-coated probe due to coating layer of about 35 nm, and the resulting larger contact area between probe and surface during force spectroscopy. The value of *n*_av_ of the specific interaction was 1.1–1.5 for the Si probe, demonstrating a successful single-molecule modification. Similar values of *n*_av_ were obtained for the other three Si probes with a maximum value of 2.1. Meanwhile, a larger value of *n*_av_ = 3.5–4.4 was obtained for the Au-coated probe, which again can be attributed to the larger tip radius resulting in more ‘click’ reactions during modification.

**Figure 4:**
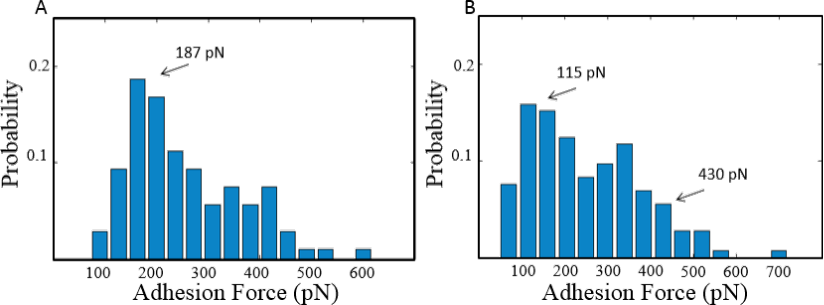
Distribution of bond rupture forces for (A) Si probes (B) Au-coated probes. The most probable force for Si probes was 187 pN, and that for Au-coated probes was 115 pN. In (B), a significant drop of probability was observed for forces larger than 430 pN.

The distribution of the bond-rupture force also supported the above Poisson statistical analysis. A total of 212 measured forces from the Si probes and 145 from the Au-coated probes were plotted in Figure 4. The most probable force for the Si probes was centered at 187 pN, which agreed well with the sum of *F*_i_ and *F*_0_ in previous Poisson analysis (183 pN for Si probes). This also indicated that *F*_i_ and *F*_0_ had similar probabilities when the probe interacted with the surface. Therefore, it was difficult to distinguish them from the histogram, as shown in Figure 4A. On the other hand, the Au-coated probes showed a much wider distribution with the most probable force centered at 115 pN, as shown in Figure 4B. The value agreed with the *F*_0_ of an Au-coated probe (111 pN). Considering the similar probability of *F*_i_ and *F*_0_, the forces obtained from Au-coated probes should be evenly distributed in the range from *F*_0_ to *F*_0_ + *n*_av_*F*_i_. A significant drop of probability was observed for forces larger than 430 pN. Therefore, it was reasonable to take this threshold value as the upper limit of force range *F*_0_ + *n*_av_*F*_i_. The resulting value of *n*_av_ was 3.3, which also agreed well with the theoretical analysis of *n*_av_ = 3.5–4.4.

**Figure 5:**
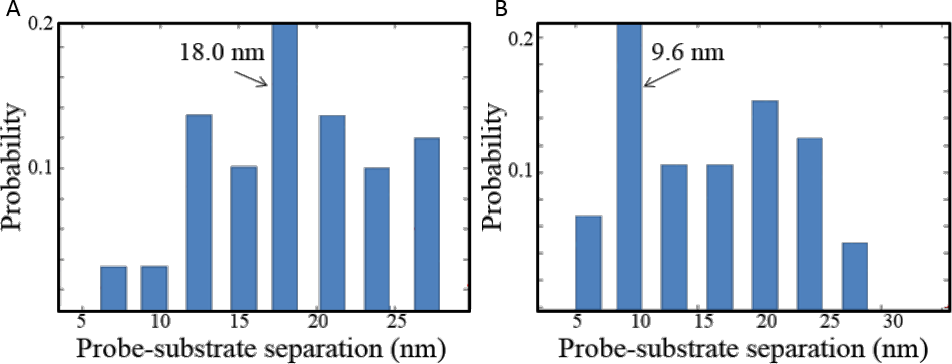
Distribution of probe-substrate separation where maximum adhesion force (bond rupture event) was recorded. (A) The distribution for the Si probe and (B) the distribution for the Au-coated probes.

The separation between probe and surface when the bond rupture occurred was also summarized and is shown in Figure 5. The PEG molecules on the glass surface had different lateral distances to the tip, and the terminal amino groups had an equal probability to interact with the carboxylic groups on the probe. Therefore, a uniform distribution was expected in the histogram of the probe–sample separation at which specific bond rupture occurred. For both (A) the Si probe and (B) the Au-coated probe, the separation distributions were relatively uniform. The higher probability at 18.0 nm for the Si probe and 9.6 nm for the Au-coated probe were probably due to higher density of carboxylic groups on the surface.

## Conclusion

In summary, we have developed a new modification method for single-molecule probes that takes advantage of a highly efficient ‘click’ reaction. As a demonstration, carboxylic acid-terminated azide molecules were successfully ‘clicked’ onto an alkyne-modified probe when the probe was in contact with the Cu substrate, which acts as catalyst. This ‘click’ reaction could only occur within the small contact area, resulting in a highly defined probe functionality. Force spectroscopy analysis along with control experiments proved that a single carboxylic group was successfully modified onto the AFM probe. We believe this method would benefit AFM-based SMFS applications and improve the reproducibility of these studies.

## Experimental

### Materials

The Fmoc-NH-PEG-NHS (*M*_p_ = 3219 Da) was purchased from Rapp Polymere GmbH. The silicon AFM probes (MSNL-C) were purchased from Bruker Nano, Inc. The nominal spring constant is 10 pN/nm and tip curvature is 2 nm. The Au-coated AFM probes (CSG10/Au) were gifts from NT-MDT with 2.5 nm adhesive Ti layer and 35 nm Au layer, the nominal spring constant is 110 pN/nm and tip curvature is 35 nm. The aminopropylsilane-coated glass slides (C18-5131-M20) were purchased from Thermo Scientific. All other chemicals including isopropanol are of reagent grade from Sigma-Aldrich. AFM experiments were carried out on a Bruker Dimension ICON AFM system.

#### Preparation of AFM probes

**Amino-functionalization of Si probes:** Si probes were functionalized with amino groups according to a previously reported procedure [26–27]. Briefly, the probes were soaked in chloroform for 10 min, and then, the solvent was removed and the process was repeated. After drying under argon flow, the probes were soaked in a freshly prepared piranha solution (30 vol % H_2_O_2_/H_2_SO_4_, 1:3, v/v) for 30 min. After rinsing with water three times, the probes were washed with methanol (two times) and chloroform (two times) and dried in a stream of argon. For amination, the probes were suspended above (3 cm) a solution of 8% (v/v) (3-aminopropyl)triethoxysilane (APTES) in toluene in a desiccator filled with dry nitrogen for 3 h (step a in Figure S1, Supporting Information File 1). Finally, the probes were treated on a plate at 100 °C in air for 10 min.

**Amino-functionalization of Au-coated probes:** The amino-functionalized Au-coated probes were prepared by incubating in a 2 mM solution of 2-aminoethanethiol in EtOH overnight (step a in Figure S2, Supporting Information File 1) [28–29]. The probes were washed with water, ethanol and chloroform, and dried in a stream of argon.

**Alkynylation of amino-functionalized probes:** The amino-functionalized silicon and Au-coated probes were incubated in a solution (4 mL) containing 4-pentynoic acid (1.25 mM), acetic acid (8.75 mM), *O*-benzotriazole-*N*,*N*,*N*’,*N*’-tetramethyluronium hexafluorophosphate (HBTU) (10 mM) and *N*,*N*-diisopropylethylamine (DIPEA) (10mM), in dimethylformamide (DMF) for 3 h (step b in Figure S1 and Figure S2, Supporting Information File 1). The probes were washed with DMF, CHCl_3_ and CH_3_OH, and dried in a stream of argon.

#### Preparation of amino-PEG-substrates

The aminopropylsilane-coated glass slides were washed with chloroform three times and dried in a stream of argon. They were then incubated in a solution of Fmoc-NH-PEG-NHS (5 mg) and triethylamine (TEA) (5 µL) in CHCl_3_ (1 mL) at rt under argon overnight (step a in Figure S3, Supporting Information File 1). After washing with chloroform and MeOH, and drying with a stream of argon, the Fmoc protecting groups were removed with 20% piperidine (v/v in DMF) for 30 min (step b in Figure S3, Supporting Information File 1). The slides were washed with DMF, CHCl_3_, MeOH, and dried in a stream of argon. After each modification process, the probe and substrate surfaces were characterized with FTIR and contact angle measurements. As shown in Figure S4 and Table S1 in Supporting Information File 1.

#### AFM force spectroscopy experiment

**Single-molecule probe modification:** The alkyne-modified probe was engaged to the Cu substrate in contact mode with 1 nN force in 0.05 M azide solution. The average surface roughness *R*_a_ of the Cu surface was 1.33 ± 0.24 nm determined through AFM topography (shown in Figure S8, Supporting Information File 1). The probe was kept in contact with the substrate for 5 to 10 s giving enough time for the Cu-catalyzed ‘click’ reaction to take place (step c in Figure S1 and Figure S2, Supporting Information File 1). Finally, the probe was retracted and rinsed thoroughly with DI water. The three probes for the control experiments were prepared under the same conditions except that one element, either Cu, alkyne or azide, was lacking. The typical force curves obtained with the control probes are shown in Figure S6 (Supporting Information File 1).

**Characterization of single-molecule modified probes:** In order to verify the result of the modification, the probe was ramped over a glass slide with a monolayer of amino-terminated poly(ethylene glycol) (PEG) in isopropanol. The amino groups should form hydrogen bonds with the carboxylic acid group on the probe in the contacting period during ramping. When the probe is retracted, the force to break the hydrogen bonds should be detectable. The force spectroscopy experiments were carried out in contact mode at room temperature in isopropanol. The spring constant of the AFM probes were calibrated by using the thermal noise method [30]. The measured values ranged from 15 to 40 pN/nm. Each probe was ramped at five different locations on the substrate, and more than 200 force curves were recorded at each location resulting in more than 1000 force curves per probe. For each ramp, the probes were first brought into contact with the substrate at a contact force of ca. 200 pN for 1 s, and then retracted. The approaching and retracting speed was 300 nm/s. Typical force curves are shown in Figure S7 (Supporting Information File 1). The force curves with either no obvious adhesion events or only non-specific adhesion at zero separation were also shown. Note that sometimes the specific and non-specific interactions co-existed, as in Figure S7D (Supporting Information File 1). The control experiments were carried out under the same conditions.

## Supporting Information

File 1 Additional experimental data.
