# Supplementary material for: Modification of a single-molecule AFM probe with highly defined surface functionality
Source: Beilstein J Nanotechnol. 2014 Nov 14;5:2122–8. doi: 10.3762/bjnano.5.221 (PMC4273215; doi:10.3762/bjnano.5.221)
Supplement: File 1 — Additional experimental data. [file Beilstein_J_Nanotechnol-05-2122-s001.pdf]

Supporting Information

for

Modification of a single-molecule AFM probe with highly defined surface functionality

Fei Long[§1], Bin Cao[§2], Ashok Khanal[2], Shiyue Fang*[2], and Reza Shahbazian-Yassar*[1]

Address: [1]Department of Mechanical Engineering-Engineering Mechanics, Michigan Technological University, 1400 Townsend Drive, Houghton, Michigan, USA and [2]Department of Chemistry, Michigan Technological University, 1400 Townsend Drive, Houghton, Michigan, USA

Email: Shiyue Fang* - Shifang@mtu.edu; Reza Shahbazian-Yassar* - reza@mtu.edu

[*]Corresponding author

[§]These authors contributed equally to this work.

Additional experimental data

Preparation of AFM probes

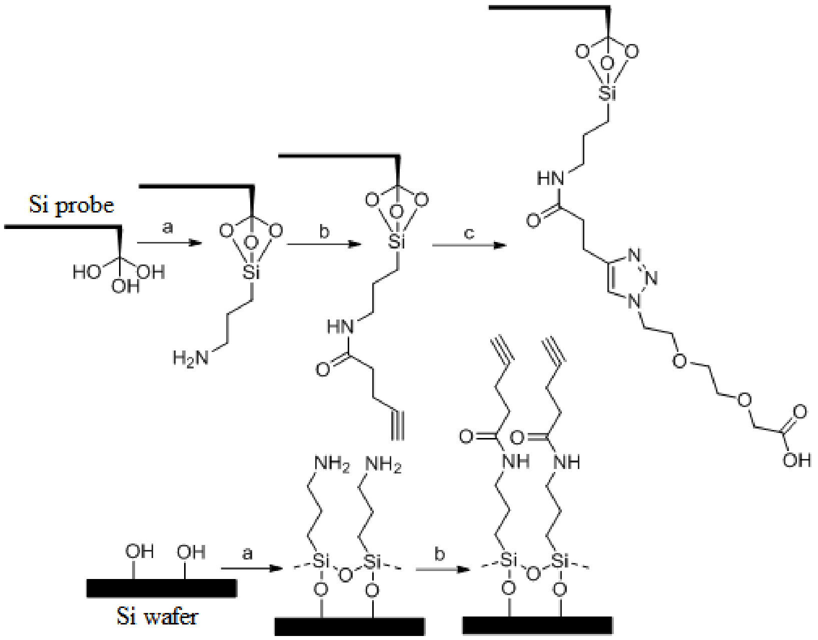

Figure S1: Si probe and Si wafer modification. (a) 3-amino-propyltriethoxysilane (APTES), 3 hours; (b) 4-pentynoic acid and acetic acid (1:7)/ N,N-diisopropylethylamine (DIPEA)/ O-benzotriazole-N,N,N',N'-tetramethyl-uronium-hexafluoro-phosphate (HBTU), dimethylformamide (DMF), 3 hours; (c) Ramping in a solution containing 2-(2-(2-azidoethoxy)ethoxy)acetic acid (compound **3**, 0.05M) in EtOH over Cu film.

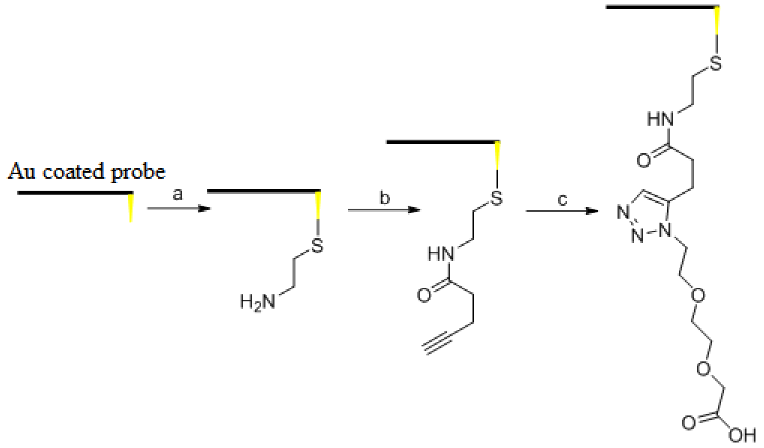

Figure S2: Au coated probe modification. (a) 2-aminoethanethiol, EtOH, overnight; (b) 4-pentynoic acid and acetic acid (1:7)/DIPEA/HBTU, DMF, 3 hours; (c) Ramping in a solution containing 2-(2-(2-azidoethoxy)ethoxy)acetic acid (compound **3**, 0.05M) in EtOH over Cu film.

Preparation of amino-PEG-substrates

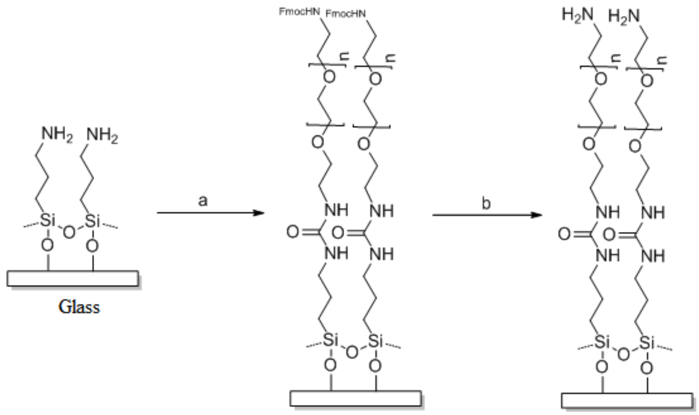

Figure S3: Substrate modification. (a) Fmoc-NH-PEG-NHS, triethylamine (Et_3N), chloroform ($CHCl_3$), overnight; (b) 20% piperidine in DMF, 30min.

Characterization of functionalized probes and substrates

FTIR spectra and contact angles were measured after each step to monitor the probe and substrate modification process. Because these techniques are not applicable to AFM probes, a silicon wafer was modified in parallel with the probes under exactly the same conditions and characterized [1,2].

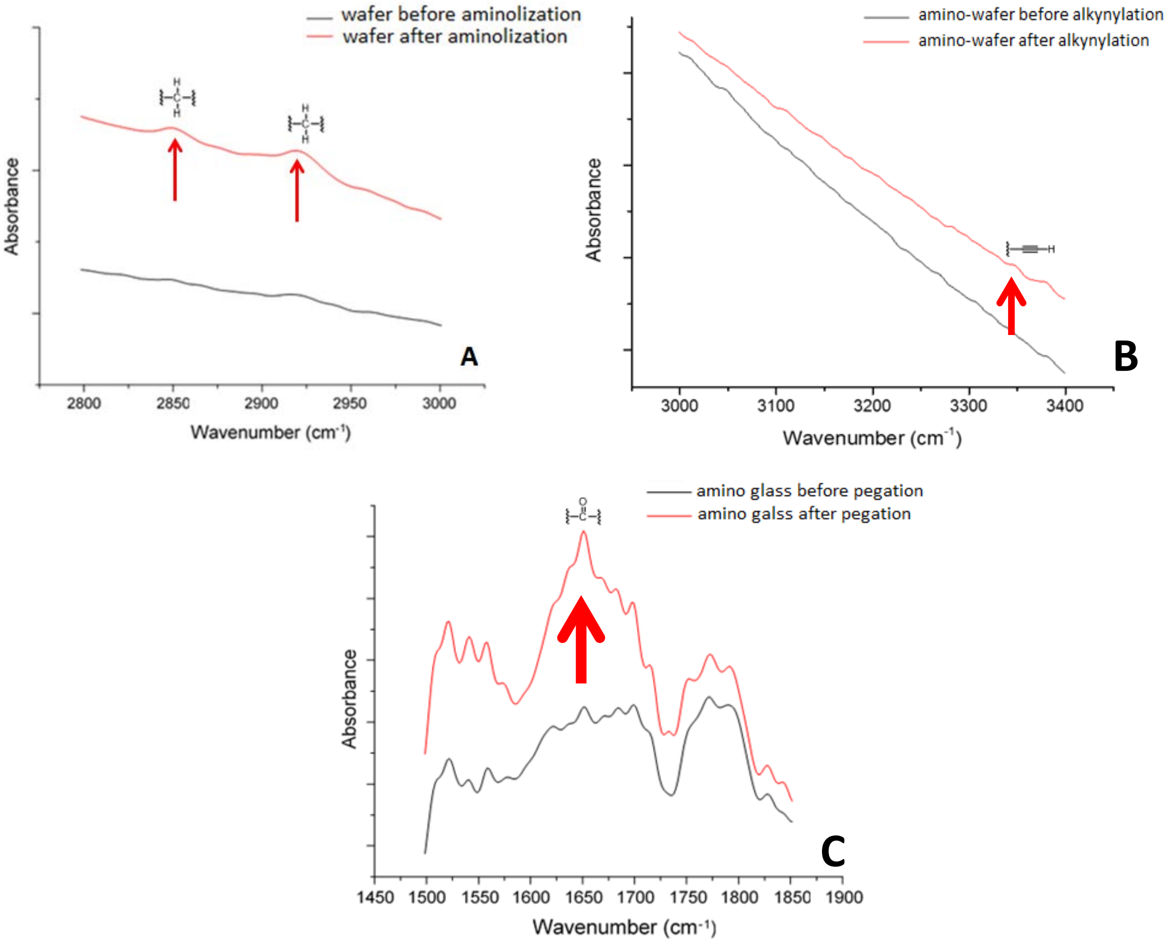

Figure S4: ATR-FTIR spectra of silicon wafer and substrate. (A) Silicon wafer before and after amino-functionalization; C-H stretching signals were observed at ~2850 cm^{-1} and ~2925 cm^{-1} for the latter; (B) Amino-wafer before and after alkynylation; alkyne C-H stretching signal at ~3320 cm^{-1} was observable for the latter; (C) Amino-glass substrate before and after NH_2-PEG modification; the signal for urea was observed at ~1650 cm^{-1}.

Infrared spectra were obtained on an Attenuated Total Reflection-Fourier Transform Infrared (ATR-FTIR) spectrometer. In all cases, absorbance spectra display changes in chemical features of the silicon sample and glass sample due to the functionalization process. The spectra shown in Figure S4 were averages of 100 scans obtained at 8 cm^{-1} resolution. Two peaks (~2850 cm^{-1} and ~2925 cm^{-1}) due to C-H stretch were observed for amino wafer (Figure S4, A). After the amino wafer was coupled with 4-pentynoic acid, one peak at ~3320 cm^{-1} corresponding to a terminal alkyne C-H stretch was observed (Figure S4, B). The pegated aminopropylsilane coated glass

slide contains a urea bond; the signal of carbonyl stretching was observed at ~1650 cm^{-1} (Figure S4, C).

Contact angles were measured using the sessile droplet method at RT in air. Measurements were repeated three times for each sample and averages are reported. The contact angle of water on the surface decreased sharply from 39.0° (Table 1) for silicon wafer to 16.8° for silicon wafer cleaned with piranha solution. After amino-functionalization, the contact angle changed from 16.8° to 46.5°, which is consistent with data reported in the literature,[3] indicating formation of amino monolayer. Treating the APTES monolayer with acetic acid and 4-pentynoic acid (7:1) in the presence of HBTU increased the contact angle to 49.4° indicating the success of the coupling reactions.

Table S1: Contact angles of surfaces before and after each modification process.

Surfaces	contact angle (deg)
Silicon wafer	39.0±0.2
Silicon wafer cleaned with piranha solution	16.8±1.4
Amino wafer	46.5±0.2
Alkynyl wafer	49.4±0.2

Synthesis of 2-(2-(2-azidoethoxy)ethoxy)ethanol (2) [4]

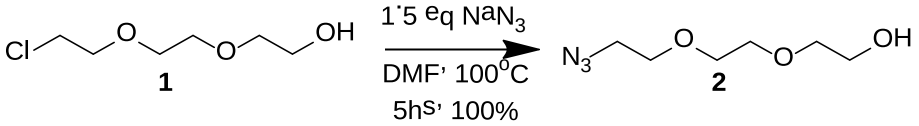

2-(2-(2-Chloroethoxy)ethoxy)ethanol (2.5 g, 14.83 mmol) was added to a suspension of NaN_3 (1.45 g, 22.3 mmol) in dry DMF (25 ml). The resulting yellow mixture was stirred at 100 °C under a nitrogen atmosphere. The reaction progress was monitored with TLC (CH_2Cl_2-MeOH, 9:1, v/v). After 5 hours, the mixture was cooled to RT, and filtered through Celite®545 to remove sodium salts. The filtrate was evaporated to dryness. The residue was purified by flash column chromatography(SiO_2, CH_2Cl_2-MeOH) to give compound **2** as a colorless oil (2.6 g, 100%) : ^{1}H NMR (400 MHz, $CDCl_3$): δ 3.09 (brs, 1H, OH), 3.22 (t, J = 4.0 Hz, 2H), 3.55-3.41 (m, 10H); ^{13}C NMR (100 MHz, $CDCl_3$): δ 72.7, 70.6, 70.4, 70.0, 61.6, 50.7.

Synthesis of 2-(2-(2-azidoethoxy)ethoxy)acetic acid (3) [4]

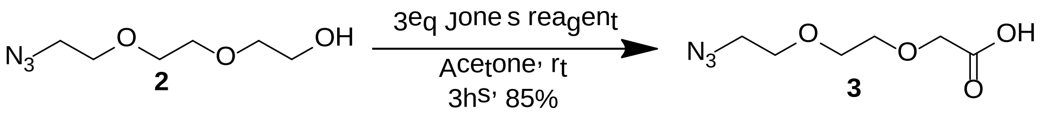

2-(2-(2-Azidoethoxy)ethoxy)ethanol (2.56 g, 14.6 mmol) was dissolved in acetone (100 mL) and the solution was cooled to 0 °C. Freshly prepared 3 M Jones' reagent (14.63 mL) was added dropwise. A green precipitate was immediately formed. The reaction mixture was stirred at RT. The reaction progress was monitored with TLC (CH_2Cl_2-MeOH, 9:1, v/v). After 3 hours, excess propan-2-ol (ca. 7 mL) was added to quench the reaction. The mixture was stirred for 15 min, and acetone (100 mL) was added. The green precipitate of Cr(III) salts was removed by filtration through Celite® 545. The filtrate was evaporated to dryness. The oily residue was immediately

purified with flash column chromatography (SiO_2,0-5% MeOH in CH_2Cl_2) giving compound **3** as a yellow oil (2.35 g, 85%): ^{1}H NMR (400 MHz, $CDCl_3$): δ 3.33 (t, J = 4.0 Hz, 2H), 3.59-3.69 (m, 6H), 4.13 (s, 2H), 9.82 (brs, 1H, COOH); ^{13}C NMR (100 MHz, $CDCl_3$): δ 50.7, 68.5, 70.2, 70.7, 71.2, 174.9.

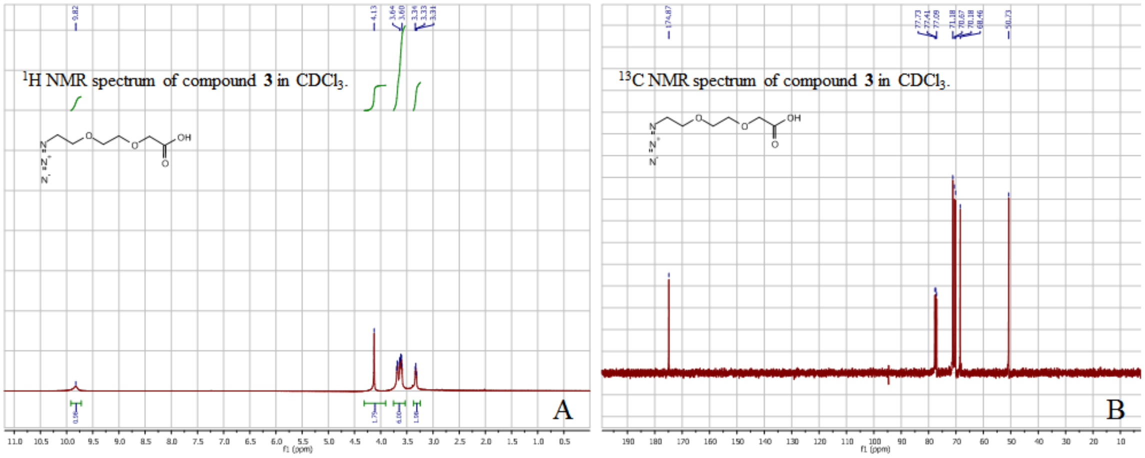

Figure S5: (A) ^{13}C and (B) ^{1}H NMR spectra of compound **3** in $CDCl_3$.

AFM force spectroscopy experiment

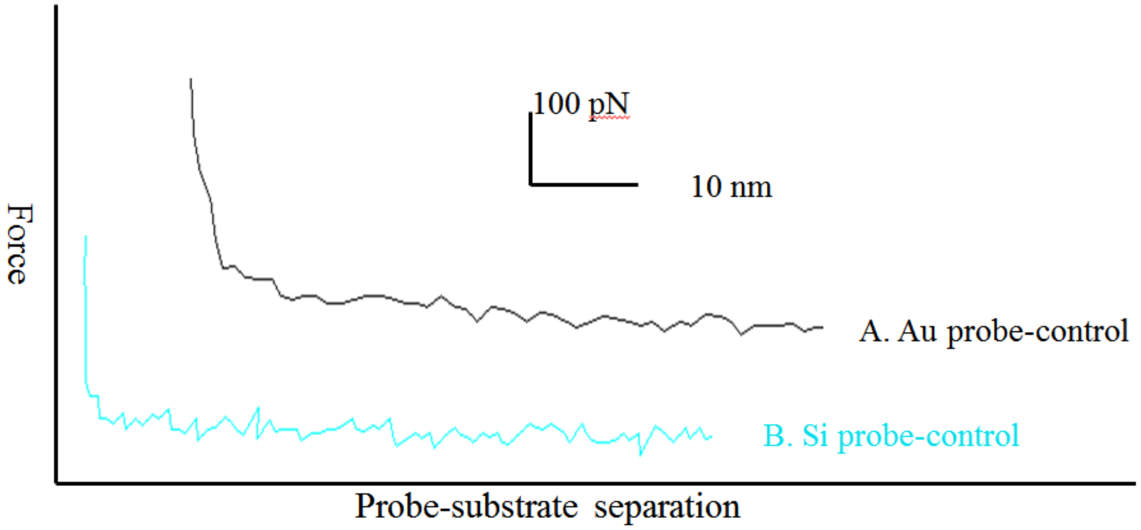

Figure S6: Typical force curves for (A) Au-coated control probe; (B) Si control probe.

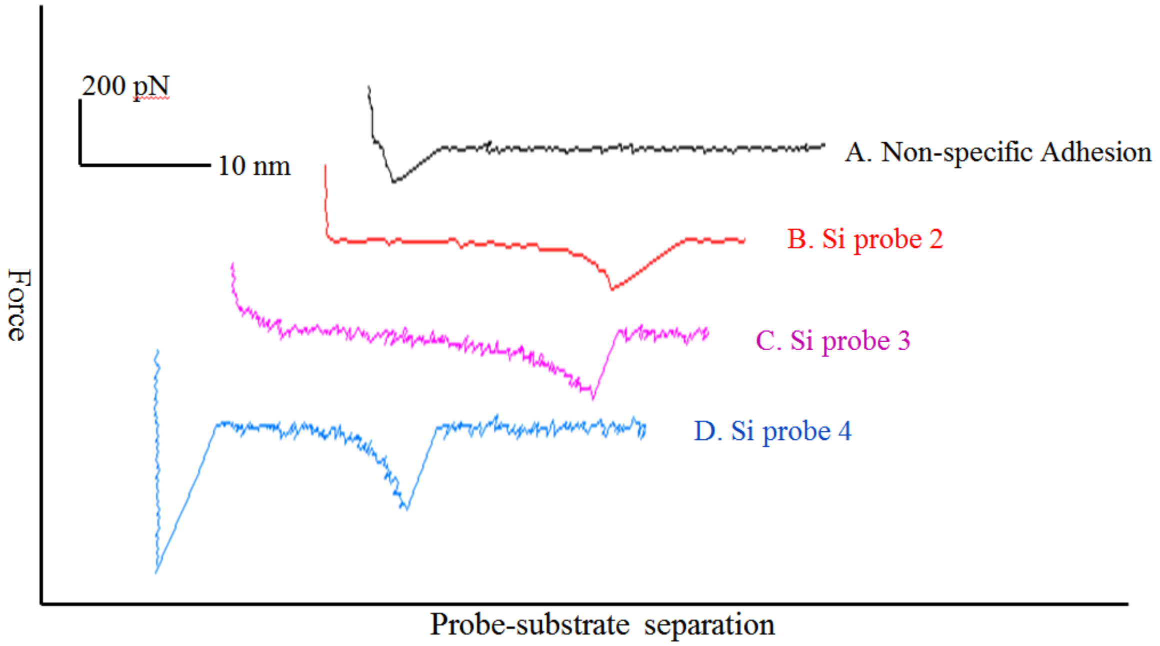

Figure S7: Typical force curves for (A) Non-specific adhesion at zero separation; (B) ~ (D) Specific adhesion for the other 3 Si probes. Note that in force curve (D), specific and non-specific adhesion co-existed.

AFM imaging of Cu-coated substrate for roughness analysis

Cu-coated glass substrates were imaged by Tapping mode in ambient condition. Bruker RTESPA cantilevers were used (nominal spring constant 42 N/m, resonant frequency 300 kHz), and typical topography is shown in Figure S10.

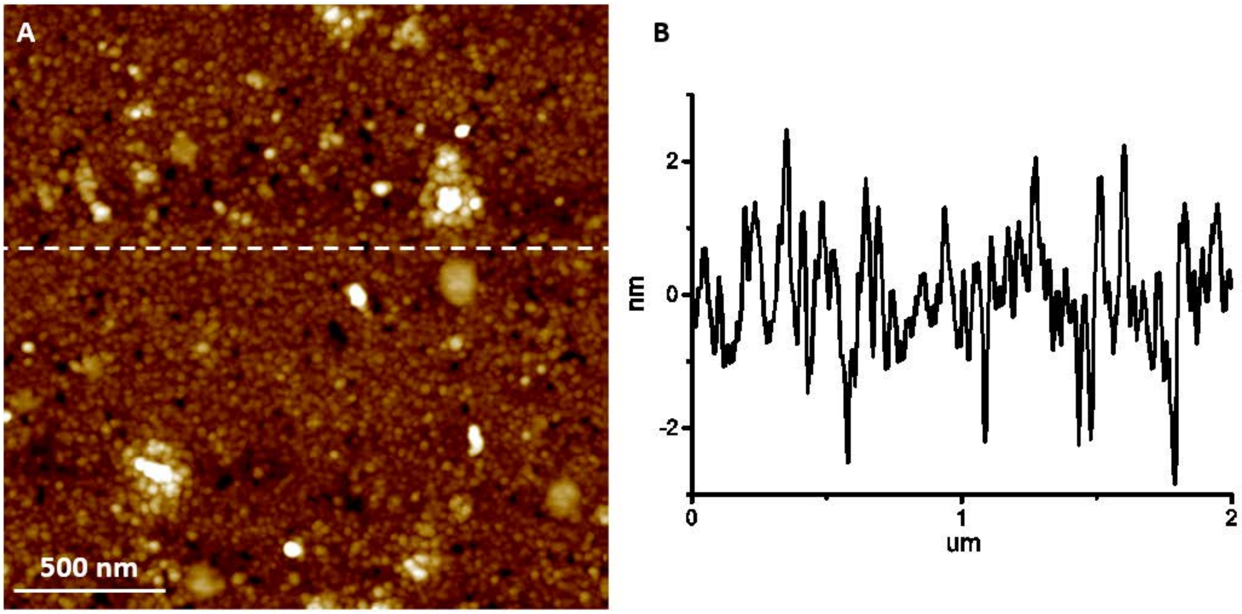

Figure S8: (A) Typical surface topography of Cu-coated glass substrate. (B) Cross section profile along the dashed line in (A).

References

[1]. Razvag, Y.; Gutkin, V.; Reches, M. *Langmuir* **2013,** *29*, 10102–10109.

[2]. Senapati, S.; Manna, S.; Lindsay, S.; Zhang P. *Langmuir* **2013,** *29*, 14622–14630.

[3]. Zeng, X.; Xu, G.; Gao, Y.; An, Y. *J. Phys. Chem. B* **2011,** *115*, 450–454.

[4]. Clave, G.; Boutal, H.; Hoang, A.; Perraut, F.; Volland, H.; Renard, P. Y.; Romieu, A. *Org. Biomol. Chem.* **2008,** *6*, 3065–3078.
